# Sibling Death in the Family and Dementia Risk

**DOI:** 10.1093/geroni/igaa057.3182

**Published:** 2020-12-16

**Authors:** Hyungmin Cha, Patricia Thomas, Debra Umberson

**Affiliations:** 1 The University of Texas at Austin, Austin, Texas, United States; 2 Purdue University, West Lafayette, Indiana, United States; 3 University of Texas at Austin, Austin, Texas, United States

## Abstract

Growing evidence points to the role of stress in contributing to dementia risk, and experiencing the death of a family member is a particularly stressful life event. Sibling relationships are typically life-long relationships and the death of a sibling is likely to be a stressful event in the life course; however, there is little research illuminating the possible consequences of sibling loss for dementia risk. This study considers whether experiencing the death of a sibling before midlife is associated with subsequent dementia risk and how such losses, which are more common for Black and Hispanic than for White populations, may add to racial/ethnic disparities in dementia risk. We use discrete-time event history models to predict dementia incidence among 9,590 non-Hispanic white, 1,669 non-Hispanic black, and 1,109 Hispanic respondents from the Health and Retirement Study, 2000-2014. Losing a sibling during the observation period is associated with increased risk for later dementia. The death of a sibling is robust to the inclusion of a variety of biosocial factors that contribute to subsequent dementia risk. The death of a sibling is a life course event with consequences that appear to increase dementia risk for Black and Hispanic older adults, and this increased risk is explained by biosocial processes likely activated by bereavement. However, Black and Hispanic Americans are further disadvantaged in that they are more likely than White Americans to experience the death of a sibling, and such losses add to the already substantial racial disadvantage in dementia risk.

